# Multifunctional Ionene Liquid Crystal Elastomers

**DOI:** 10.1021/acsami.5c08503

**Published:** 2025-07-17

**Authors:** Zachary Kuzel, Arul Clement, Mohsen Tabrizi, Abdullah AboHussien, Sivakumar Irla, Hassan Beheshti Seresht, Youngjae Chun, Stephanie Tristram-Nagle, Qihan Liu, M. Ravi Shankar

**Affiliations:** † Department of Industrial Engineering Swanson School of Engineering, 6614 University of Pittsburgh, 3700 O’Hara Street, Pittsburgh, Pennsylvania 15261, United States; ‡ Department of Bioengineering Swanson School of Engineering, University of Pittsburgh, 3700 O’Hara Street, Pittsburgh, Pennsylvania 15261, United States; § McGowan Institute for Regenerative Medicine, University of Pittsburgh, 450 Technology Drive, Pittsburgh, Pennsylvania 15219, United States; ∥ Biological Physics Group, Physics Department, Carnegie Mellon University, 5000 Forbes Avenue, Pittsburgh, Pennsylvania 15213, United States; ⊥ Department of Mechanical Engineering and Materials Science Swanson School of Engineering, University of Pittsburgh, 3700 O’Hara Street, Pittsburgh, Pennsylvania 15261, United States

**Keywords:** ionic, LCE, adhesion, actuation, self-sensing, soft elasticity

## Abstract

Incorporating ionic species into the backbone of liquid crystalline elastomers offers a template for tailoring thermomechanical and electromechanical properties. Thermotropic ionene liquid crystalline elastomers (iLCEs) containing imidazolium-based cationic groups are capable of work-dense (∼14 J/kg), large-strain (>30%) actuation at modest temperatures (∼40 °C). Furthermore, the constitutive behavior of iLCE is modulated by ionic liquid (IL) dopants, which magnify the large strain deformability (>600%), modulate pressure-sensitive adhesion, and enable strain sensing over ∼100% strain at a constant stress defined by its soft elastic plateau. The nascent electronic conductivity of iLCE is sensitive to temperature, which unlocks a route for sustaining actuation cycles by gating the actinic stimulus using materially embodied sensing. iLCEs are also capable of athermal electromechanical actuation. Ion migration at low voltages (<3 V) in iLCEs with anisotropic molecular order produces bending strains that compete favorably against traditional ionic actuators. This responsiveness is modulated by the structure and alignment of the nematic axis with respect to the applied electrical fields. The ability to modulate the electromechanical coupling in iLCEs on top of the thermomechanical properties traditionally derived from liquid crystallinity enables a motif for assimilating an array of multifunctional properties.

## Graphical abstract

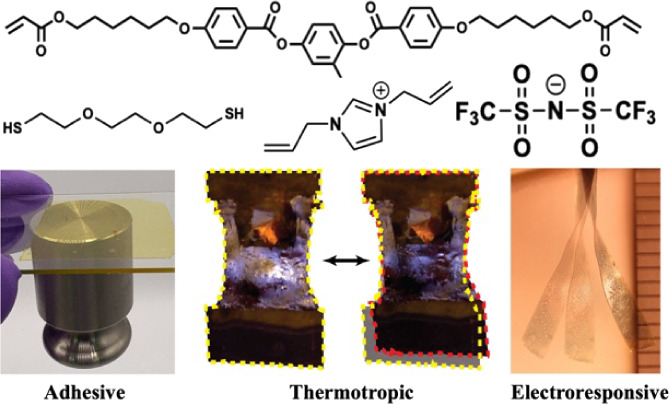

## 1 Introduction

The pursuit of high-performance materials that embody multiple functionalities presents approaches for designing soft robots,^1^ active metastructures,^2–4^ self-assembling architectures,^5,6^ and adaptive biomedical systems.^7^ Materially embodied mechanical actuation, energy transduction, state sensing, and self-regulation will transform design spaces across application areas. Given that functionality is dictated by form, the structural mutability of soft actuators presents an ideal platform for realizing highly tunable functionalities. Liquid crystalline polymers have emerged as a platform for programming structural evolution within their microstructure.^8^ These materials have been instantiated in liquid crystalline elastomers (LCEs),^9–11^ gels,^12^ composites of LCE with liquid metal (LM),^13–15^ and liquid crystalline glassy networks.^16^ A distinguishing characteristic of these materials is their ability to generate large displacements derived from blueprinted molecular order, driven by external stimuli such as thermal energy,^17^ light,^18^ electric fields,^19^ and pH change.^20^ These materials have found favor in soft robotic designs,^21,22^ with implications for biomedical devices,^23^ microbotics,^24,25^ and unlocking novel motifs for assimilating control via material design.^26^ Soft actuators are particularly attractive for integration with biological systems due to their morphability, while minimizing mismatch in moduli with biological matrices.^27,28^ Here, the ability to assimilate self-sensing^29,30^ with mechanical adaptivity can refine functional design spaces (e.g., biomedical, microrobotic, and optics) using compact form factors.^31,32^

LCEs are capable of remarkable thermal shape memory and actuation. Thermotropic LCEs embody a work density comparable to that of muscle tissue when subjected to a temperature rise, while generating strains >50%.^14,31^ Breakthroughs within the framework of additive manufacturing have enabled their implementation in complex geometries, where mesogen alignment can be voxelated using shear forces or magnetic fields during 3D printing.^13,33–36^ Traditionally, films were prepared with monodomain alignment, resulting in uniaxial deformation. Photoalignment techniques allow for preprogrammed local alignment within the film, where complex deformation can be blueprinted.^37,38^ The synergy of geometric complexity coupled with programmable local (voxelated) mesogen alignment is a powerful design tool. The ability to power LCEs with electrical actuation is key to utilizing their responsiveness in application spaces. Limitations arise in delivering thermal stimuli within a reliable control framework. This is especially a challenge in hydrated environments or those characterized by large ambient thermal fluctuations or thermal dissipation. Efforts have exploited eutectic gallium indium (eGaIn) liquid metal (LM) electrodes that are composited with LCEs, where Joule heating was used to power actuation at low voltages (<5 V)^14^. With suitable optimization and thermal isolation, work-dense actuation at ∼100 mW-scale power inputs has been accomplished.

An athermal approach to soft actuation using electrical power emerges with ionic-polymer composite (IPC) films that show robust cantilever beam bending using low-intensity electric fields emerging from the application of 1–5 V.^39–42^ From a micromechanical perspective, the bending of the films is the result of a volume displacement driven by ion migration. Deformable electrodes such as single-walled carbon nanotubes, poly­(3,4-ethylenedioxythiophene)–poly­(styrenesulfonate) (PEDOT:PSS), and graphene have been used to power this bending. These efforts grow from advances in ionic-polymer–metal composites (IPMC), where noble metals such as Pt have been used as electrodes in ionic polymers such as Nafion.^43–46^ Optimization of the polymer structure (e.g., nanostructured triblock polymers^47^) and modulation of the mobility of ionic species (e.g., zwitterions^48^) offer routes for optimizing actuation profiles. Another study incorporated ionic liquid (IL) into traditional LCE networks, resulting in electro-responsive LCEs.^49^ Another approach explored porous LCEs with infused ionic liquid (PLCE-IL), where the material’s resistance evolved in response to deformation.^9^

Offset from these explorations, ionenes composed of ionic moieties embedded in the backbone of a polymer network have attracted significant attention. Viologen-based polymers containing 4,4′-bipyridyl groups in the backbone show liquid crystalline behavior, in combination with electrical conductivity and electro/photochromic responses.^50^ They have demonstrated thermotropic responses and fluorescence.^51^ Imidazolium-based ionenes have been explored for gas separation with remarkable specificity.^52^ These ionenes are also amenable to the synthesis of block copolymers to engineer tailored micro/nanophases.^53^ Self-organization of imidazolium salts into liquid crystalline phases is a potent route for engineering ion transport with high degrees of anisotropy.^54^ The ability to organize these moieties in polymer matrices for applications in energy, biomedical, and engineering applications is noteworthy.^55^

Here, we demonstrate multifunctional responses that emerge from the incorporation of imidazolium-based cations within the backbone of a liquid crystalline elastomer by using a scalable synthesis framework that relies on the Michael addition reaction. The thiol–acrylate oligomerization method, which was previously used to create aligned (but apolar) nematic elastomers, was adapted here to incorporate the imidazolium moieties. The resulting oligomers allow for their alignment by mechanical deformation in a manner reminiscent of the Finkelmann’s method, following which they can be cross-linked into elastomers. iLCE presents thermotropic responses characterized by work-dense actuation profiles. With increasing temperatures, tens of percent actuation strain becomes feasible. The results illustrate thermotropic actuation in IL-doped iLCE at ∼40 ^ο^C, which is within a biocompatible temperature range. Doping iLCEs with IL is shown to offer pathways for controlling the constitutive behavior of iLCE compositions, including their toughness and soft elastic plateau. This modulation of the mechanical response also holds implications for utilization of iLCE as adhesives, which is magnified by doping with IL. This offers opportunities for exploiting observations of pressure sensitive and thermally switchable adhesion using LCE.^56^ The thermotropic actuation of iLCEs is capable of self-reporting the actuation state due to temperature-dependent electrical resistance. A 2-fold decline in the conductivity coincides with the thermotropic actuation window. The large deformability of the iLCE within their soft elastic plateau also enables a readout of the mechanical strain over a range of ∼100% via a change in the electrical resistance without concomitant stress hardening. When doped with IL, the molecular order magnifies the actuation strains resulting from ion migration due to applied voltages. Athermal actuation strains comparable to those of state-of-the-art ionic polymers become feasible, which compare favorably against conventional ionic actuators (e.g., IPMC^57^ and polymer electrolyte systems^58^), or IL-carbon nanotube actuators.^59^ The array of multifunctional property combinations possible with iLCE can unlock system design opportunities within a tunable synthesis and manufacturing framework, with applications for soft actuators, sensors, polymer electrolytes, and biomedical applications.

## 2 Experimental Methods

A mesogenic diacrylate, 1,4-bis-[4-(6-acryloyloxyhexyloxy)­benzoyloxy]-2-methylbenzene (RM82), was purchased from a commercial source (Wilshire Technologies). The photoinitiator 2-benzyl-2-(dimethylamino)-4’-morpholinobutyrophenone (I-369), the vinyl cross-linker, 1,3,5-triallyl-1,3,5-triazine-2,4,6­(1*H*,3*H*,5*H*)-trione (TATATO) used in the control LCE samples, the thiol chain extender 2,2’-(ethylenedioxy)­diethanethiol (EDDT), the unreactive IL for doping the iLCE: 1-hexyl-3-methylimidazolium (HMIM) bis­(trifluoromethylsulfonyl)­imide (TFSI), and the conductive electrode layer PEDOT:PSS were all purchased from Sigma-Aldrich. Triethylamine (TEA), which was used as a base catalyst, was purchased from TCI Chemicals. Indium and gallium were purchased from Indium Corporation to synthesize liquid metal (LM) layers. Indium tin oxide (ITO)-covered glass slides were purchased from SPI Supplies (resistance of 70 to 100 Ω).

### 2.1 Synthesis of Imidazolium-Functionalized Cross-Linkers

The first step of the synthesis involved the preparation of 1,3-diallylimidazolium bromide (see Figure S1). To a round-bottom flask were added 1-allylimidazole (1.0 mol) and allyl bromide (1.05 mol) in an acetonitrile solution (CH_3_CN). The resulting solution was stirred for 24 h at 80 °C. Following concentration of the reaction mixture under reduced pressure, the resulting viscous liquid was washed with hexane. The product was dried in vacuum and used in the next step without purification. This product was used in the preparation of 1,3-diallylimidazolium bis­(trifluoromethylsulfonyl)­imide. A solution of 1,3-diallylimidazolium bromide (1 mol) was dissolved in distilled water and stirred. Lithium bis­(trifluoromethylsulfonyl)­imide was dissolved in distilled water. This was slowly added to the reaction mixture and stirred for 24 h at room temperature. Chloroform was added to the reaction mixture, and then the organic phase was extracted and washed with water. The organic phase was evaporated under vacuum. ^1^H NMR (CDCl_3_) δ 8.67 (s, 1H), 7.35 (s, 2H), 5.93–6.03 (m, 2H), 5.44–5.48 (m, 4H), 4.78 (d, 4H, *J* = 6.4 Hz). ^13^C NMR (CDCl_3_): 135.18, 129.24, 124.55, 123.02, 122.45, 121.36, 118.17, 114.98, 52.16. Figure S2 contains the NMR plots.

### 2.2 iLCE Synthesis

iLCE films were synthesized via thiol–acrylate and thiol–ene Michael addition reactions. The elastomeric network is initially composed of unconnected oligomers from the liquid crystalline monomer RM82, the thiol chain extender EDDT, and covalently bonded imidazolium cations. Figure 1a shows the molecular structures used in the synthesis, excluding the photoinitiator species. RM82 and EDDT, at a 1:0.9 molar ratio, are initially heated to 120 °C to melt the solid material and subsequently mixed on a rotary mixer for a few seconds. Then, the imidazolium-functionalized cross-linker IL and I-369 were incorporated to synthesize RMEDDT-IMIL. RMEDDT-IM was similarly synthesized, except that the IL was not used. RMEDDT-IL and RMEDDT were synthesized with TATATO as the cross-linking agent in lieu of the imidazolium-functionalized cross-linker. The compositions are shown in Table 1. Subsequently, the mixtures were heated and mixed again before adding 2 drops of the base catalyst TEA to initiate the oligomer reactions. The material is then transferred to a cell that is prepared by the following steps.

**Table 1:** Composition of Various Samples Explored in This Study^a^

Composition	Molar Ratios	RMEDDT-IMIL	RMEDDT-IM	RMEDDT-IL	RMEDDT
	RM82	1	1	1	1
	EDDT	0.9	0.9	0.9	0.9
	IM Cross-linker	0.4	0.4	0	0
	IL Dopant	0.07	0	0.07	0
	TATATO	0	0	0.3	0.3
Properties	*T*_ni_ (°C)	42 ± 1.3	57 ± 3.3	52 ± 2.6	63 ± 0.9
	*T*_g_ (°C)	–11 ± 2.0	–5 ± 0.4	–7 ± 0.2	1 ± 0.6
	ϵ_T,max_ (%)	–34.8 ± 1.3	–28.8 ± 0.4	–34.2 ± 1.1	–40.2 ± 1.1
	*S*_Xray_	0.44 ± 0.02	0.54 ± 0.02	0.46 ± 0.02	0.45 ± 0.03
	*V*_e_ (mol/cm^3^)	2.9 × 10^–5^ ± 5.0 × 10^–6^	1.7 × 10^–4^ ± 4.1 × 10^–5^	1.2 × 10^–4^ ± 2.1 × 10^–5^	2.2 × 10^–4^ ± 1.1 × 10^–5^

a Monomer structures are shown in Figure 1a. *T*_ni_: nominal nematic-isotropic transition temperature was characterized by measuring the temperature for the maximal thermomechanical strain sensitivity of the LCE using a method similar to that in ref 14. *T*_g_: glass transition temperature was measured via dynamic mechanical analysis. *S*_Xray_: the order parameter was measured using wide angle X-ray scattering (WAXS). ϵ_T,max_: maximum thermomechanical contractile strain generated by the material. *V*_e_: crosslink density.

**Figure 1:**
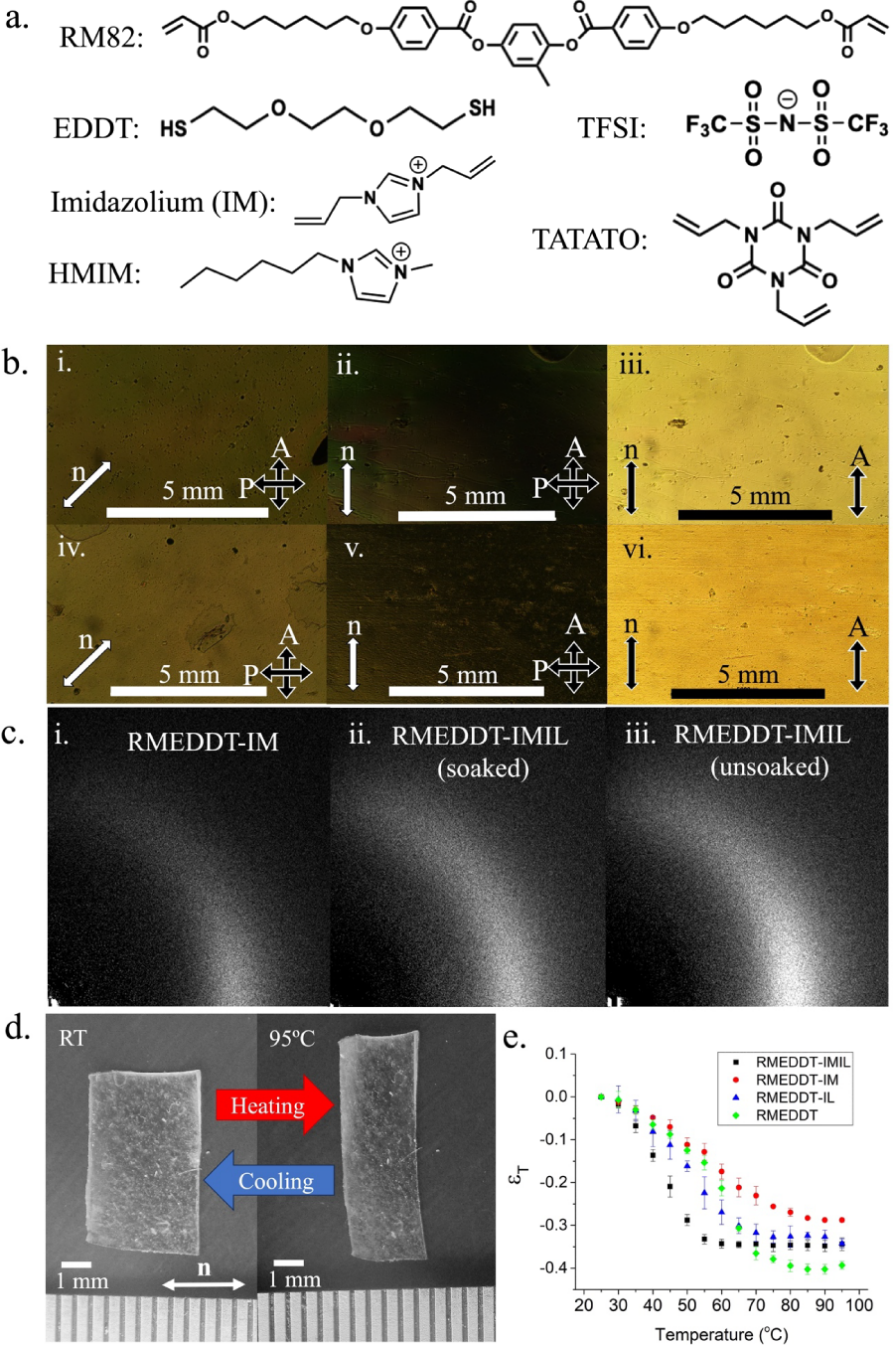
(a) Molecular structures of the monomers used in Table 1. (b) POM images of RMEDDT-IMIL (i–iii) and RMEDDT-IM (iv–vi), demonstrating the monodomain order. The orientations of the polarizer (P) and analyzer (A) with respect to the nematic director (*n*) are shown. (c) WAXS images comparing RMEDDT-IM (i) to RMEDDT-IMIL when it is unsoaked (ii) vs soaked (iii) with ionic liquid. (d) Image of the RMEDDT-IMIL film at room temperature: RT (left) versus that at 95 °C (right). (e) Thermomechanical strain response vs temperature of LCE is shown for various compositions (Table 1).

Two glass substrates were sonicated in a 2% solution of Alconox in water and rinsed with deionized water. The resin material was deposited onto one of the glass substrates, while the other was coated with Ease Release 200 (Smooth-On, Inc.) and placed on top and bound with clips, creating a cell. Spacer films ranging from 50 to 200 μm were used to control the thickness of the resulting film. The monomer mixture was introduced into the cell and heated on a hot plate at 95 °C for 2 h to allow the Michael addition reactions to occur. After heating, the oligomerized cell was lightly cross-linked while in the isotropic phase by exposing the cell to 365 nm UV light at an intensity of 8.5 mW cm^–2^ at 120 °C for 60 s. To impose a monodomain alignment, the films were stretched on a translational stage by ∼65% and held under the same UV light intensity at room temperature for 10 min to complete the polymerization. Four compositions of the iLCE were prepared by varying the ionic species present in the elastomeric network (Table 1). Additionally, polydomain iLCE samples were fabricated by omitting mechanical stretching prior to the complete photopolymerization of the film. An additional step of soaking the final film in an ionic liquid was done on RMEDDT-IMIL samples for certain experiments. Unless specifically mentioned, RMEDDT-IMIL samples refer to unsoaked films. Measurements of the gel fraction and FTIR spectroscopy were used to confirm the conversion of the monomers. The methods for both the measurement of gel fraction and FTIR are discussed in Section S3.0. The monodomain (planar) alignment of the iLCE compositions in Table 1 was confirmed using polarized optical microscopy (POM). The order parameter *S*_Xray_ of the samples was characterized via wide-angle X-ray scattering (WAXS) measurements on a Xenocs Xeuss 3.0 system. The WAXS methodology is further discussed in Section S4.0.

### 2.3 Thermomechanical Characterization

Thermotropic actuation was characterized in iLCE and LCE films by preparing 1 cm × 0.5 cm samples and tracking the displacement of the film’s length along the nematic director as a function of temperature from ambient to 95 °C. An Omega HH802U system was used to measure the temperature while the samples were placed on a hot plate. A strain–displacement relationship was used to calculate the strain of actuation: $\epsilon_{T}=\frac{L\left(T\right)}{L_{0}}-1$ , where *L*_0_ is the original length, and *L*(*T*) is the actuated length as a function of temperature (*T*). The resulting plots can be used to calculate the nominal nematic-to-isotropic transition temperature (*T*_ni_) as the one characterizing the inflection point of maximum strain sensitivity to temperature.^14^ The specific work of RMEDDT-IMIL was quantified via experiments in which the films were suspended with weights ranging from 2 to 15 g. The film was actuated with heat, and the maximum strain ε_max_ was calculated from the displacement. *L*_0_ is the initial measured length after the weight is applied. The displacement against the weight and mass of the iLCE film was used to calculate the mass-specific work density. A hot plate was positioned parallel to the vertically suspended film/weight such that the hot plate was close enough to transfer heat and induce deformation. A thermocouple was attached to the film to track the temperature during the actuation.

The storage moduli and tan δ values of the compositions in Table 1 were characterized as a function of temperature using a PerkinElmer Dynamic Mechanical Analysis (DMA) 8000 system. The maximum value for tan δ is used to identify the glass transition temperature, *T*_g_. The cross-linking density, *V*_e_, for the films was found by measuring the storage modulus (*E*′_high_) at a temperature 50 °C higher than *T*_g_, denoted as *T*_high_, and using the equation:^60^ Ve=Ehigh′3RThigh . All samples prepared for the DMA tests were conducted with the director oriented parallel to the tensile axis of the test. Stress–strain measurements were performed on a Starrett FMS500 tensile tester with a 10 N load cell and a 10 mm/min strain rate. Monodomain RMEDDT-IMIL films (80 μm thick) were prepared and cut into rectangular samples such that the director orientation had a 90°, 45°, or 0° relative to the applied tension. Polydomain samples were also studied with this setup. To study the effects of IL within the elastomer network, both a traditional tension test and a probe tack test of polydomain RMEDDT-IM were performed. The soaked and unsoaked RMEDDT-IMIL films were compared. A Shimadzu EZ-LX HS tension tester with a 500 N load cell was outfitted to perform a probe tack adhesion test using glass parallel plate probes on polydomain iLCE films with dimensions of ∼1 cm × 1 cm × 150 μm. The samples were preloaded between the probes at 5 N for 60 s before subsequently applying a fixed displacement rate of 10 mm/min. These tests characterized the “nonannealed” case. Another set of experiments involved using a heat gun to heat-treat the sample prior to the measurements of adhesive strength. This “annealed” sample was subjected to heat treatment until the glass substrate’s temperature exceeded 70 °C. Then, contact was made, and a preload of ∼5 N was maintained. Then, the probe, substrates, and the iLCE sample were allowed to cool back to room temperature. Subsequently, the test was performed at a displacement rate of 10 mm/min.

### 2.4 Electrical Characterization

The electrical resistance of the iLCE was measured as a function of temperature. For this, the iLCE film was sandwiched between two ITO-coated coverslips and connected to a Fluke 83 Multimeter. The setup was placed on a hot plate, and the resistance was measured as a function of temperature. These measurements enable a framework for assimilating self-sensing during iLCE via thermotropic actuation. Strain versus resistance was found by preparing RMEDDT-IMIL films and using the Starrett tensile system. Samples were prepared with a 90° molecular orientation with respect to the axis of the tension test. Samples with widths of 4 mm, thicknesses of 160 μm, and gauge lengths of 2.5 mm were studied. The experiment was performed in discrete steps, where a strain of 20% was imposed while measuring the force from the tension tester. The resistance of the films along the tensile axis was measured as a function of strain by using a Fluke 83 system.

### 2.5 Electromechanical Characterization

The RMEDDT-IMIL samples were cut into 10 × 4 mm rectangles and soaked with IL. The films absorbed an additional 3 mg of IL. Subsequently, they were integrated with electrode layers by spin-coating PEDOT:PSS on both sides at 1000 rpm for 30 s. Afterward, the coated film was placed on a hot plate at 50 °C for 10 min to create electrodes of ∼5 nm in thickness. To characterize the electromechanical response of iLCEs, the films were vertically suspended, and the electrodes were connected to a DC waveform generator power supply. 50 μm thick copper strips were attached to glass slides, and the contacts were made between the exposed copper and the PEDOT:PSS electrode. To explore the electromechanical response of the film from ion migration, a voltage of either 1 or 3 V was applied to the film to drive ion migration. A low current of <10 mA was observed through the circuit, eliminating the possibility of thermal effects. The bending strain of the films was calculated: $\varepsilon =\frac{2\delta t}{L^{2}+\delta^{2}}$ , where δ is the tip displacement, *L* is the length of the cantilevered section of the actuated material, and *t* is the thickness.^41^ Broadband dielectric spectroscopy was also performed on both the soaked and unsoaked RMEDDT-IMIL films using a Novo Control Concept 80. Tests were conducted in the frequency range of 1 × 10^–2^ to 1 × 10^6^ Hz to measure the DC ionic conductivities.

## 3 Results and Discussion

The integration of ionene moieties within the LCE backbone was first confirmed through FTIR and gel fraction experimentation. The FTIR plot, found in Figure S3, confirms the disappearance of the alkene moieties hitherto present in the IM monomer after the oligomer is photopolymerized. The results for both the FTIR and gel fraction characterizations are discussed in detail in [Sec sec3.1] The thermomechanical and electromechanical properties of iLCE as a function of the composition were examined. This included a characterization of the responsiveness of the iLCE to thermal and electrical stimuli, as well as the cross-coupling between electrical and mechanical responses.

### 3.1 Thermotropic Actuation of iLCE

Order–disorder transitions in cross-linked LCE occur when the sample is progressively heated past the nominal nematic–isotropic transition temperature.^61^ Figure 1b illustrates the planar, monodomain-oriented nematic order observed via polarized optical microscopy (POM). WAXS was used to track the effect of the addition of IL on the order parameter (Figure 1c). Table 1 shows that the order parameters of RMEDDT-IMIL, RMEDDT-IL, and RMEDDT were comparable to one another. Interestingly, RMEDDT-IM exhibited a higher order parameter of 0.54 ± 0.02, likely resulting from the imidazolium cross-linker’s nascent liquid crystalline nature,^55^ which stabilized the nematic state in this system. However, when RMEDDT-IMIL was soaked further with an ionic liquid (for electromechanical testing) prior to the WAXS experiment, a reduction in the order parameter was observed. This is likely a result of a weakening of the intermesogenic coupling from the IL uptake. Loss of nematic order is known to occur when LCEs are swollen in solvents.^62^ Further details on the WAXS results can be found in Section S4.1. Figure S4 contains plots of the WAXS data used to derive the order parameters.

We characterized how the effect of ionic species on liquid crystalline order modulates thermotropic responses. When subjected to heat treatment, strain generation occurs over a broad temperature range, which is a function of the macromolecular architecture and the processing conditions.^14^ Figure 1d illustrates the characteristic contractile strain along the nematic director and the expansion transverse to the director when heated above the nematic–isotropic transition temperature (*T*_ni_). The development of the thermomechanical strains as a function of temperature was used to characterize the nominal *T*_ni_ value, as illustrated in Figure 1e. It is evident that the introduction of imidazolium-functionalized cross-linkers into the backbone of the iLCE (RMEDDT-IM) leads to a lower *T*_ni_ with respect to the neat, conventional LCE (RMEDDT). Furthermore, this diminution results in a degraded actuation profile, where the RMEDDT-IM produces a contractile strain smaller than that of RMEDDT (Table 1).

The introduction of the IL into the LCE and iLCE matrices leads to a lowering of the nominal *T*_ni_.^63^ The introduction of nonmesogenic Li salts (LiTFSI) has been shown to lead to a reduction in the transition temperature.^64^ This is consistent with the behavior observed here. Compare RMEDDT vs RMEDDT-IL and RMEDDT-IM vs RMEDDT-IMIL in Table 1. RMEDDT-IL presents a lower thermomechanical actuation strain than RMEDDT. However, RMEDDT-IMIL presents an anomalous magnification of the actuation strains to −34.8 ± 1.3% in comparison to that of the IL-free RMEDDT-IM, which only contains the imidazolium-functionalized cross-linkers in the backbone (−28.8 ± 0.4%). This accompanies a reduction in the *T*_*ni*_ to ∼42 °C during the generation of these large strains. This represents an actuation temperature that is in the biocompatible range, where work-dense actuation can emerge without engendering risks from hyperthermia.^65^ Inclusion of IL into the iLCE and LCE matrix induces a softening of the modulus, as is apparent from the DMA results in Figure 2a. The IL behaves as a plasticizer^66^ that lowers the cross-link density and subsequently the glassy transition temperature while decreasing the elastic modulus. The peak in the tan δ curves (Figure 2b) was found to be reduced in the samples prepared with the IL (Figure 2b). It is noteworthy that the RMEDDT-IM presents tan δ that approaches ∼1, which points to a strategy for realizing highly dissipative LCE by incorporating imidazolium-based ionic species in the backbone. Tensile tests (Figure 2c) confirmed the plasticizing effect of the IL, where the deformability of the iLCE was magnified. The toughness of the material is defined here as the area under the stress–strain curve. Polydomain samples were used in the tension tests. RMEDDT-IMIL (unsoaked) illustrates a soft elastic plateau with fracture strains of 280 ± 30% compared to RMEDDT-IM, which fractured at a strain of 230 ± 10%. The corresponding toughness values for each composition are 1.20 ± 0.30 MJ/m^3^ and 1.70 ± 0.20 MJ/m^3^, respectively. IL dopants are known to stiffen the gel into a glassy gel when they can form ionically bonded cross-linking networks.^67^ When RMEDDT-IMIL is soaked with additional ionic liquid, it exhibits a ∼2× increase in fracture strain. The toughness, however, does not show a significant increase when the iLCE is soaked in IL, yielding a value of 1.60 ± 0.20 MJ/m^3^. RMEDDT-IM samples containing no IL show a rupture strain that is lower by a factor of ∼3×.

**Figure 2:**
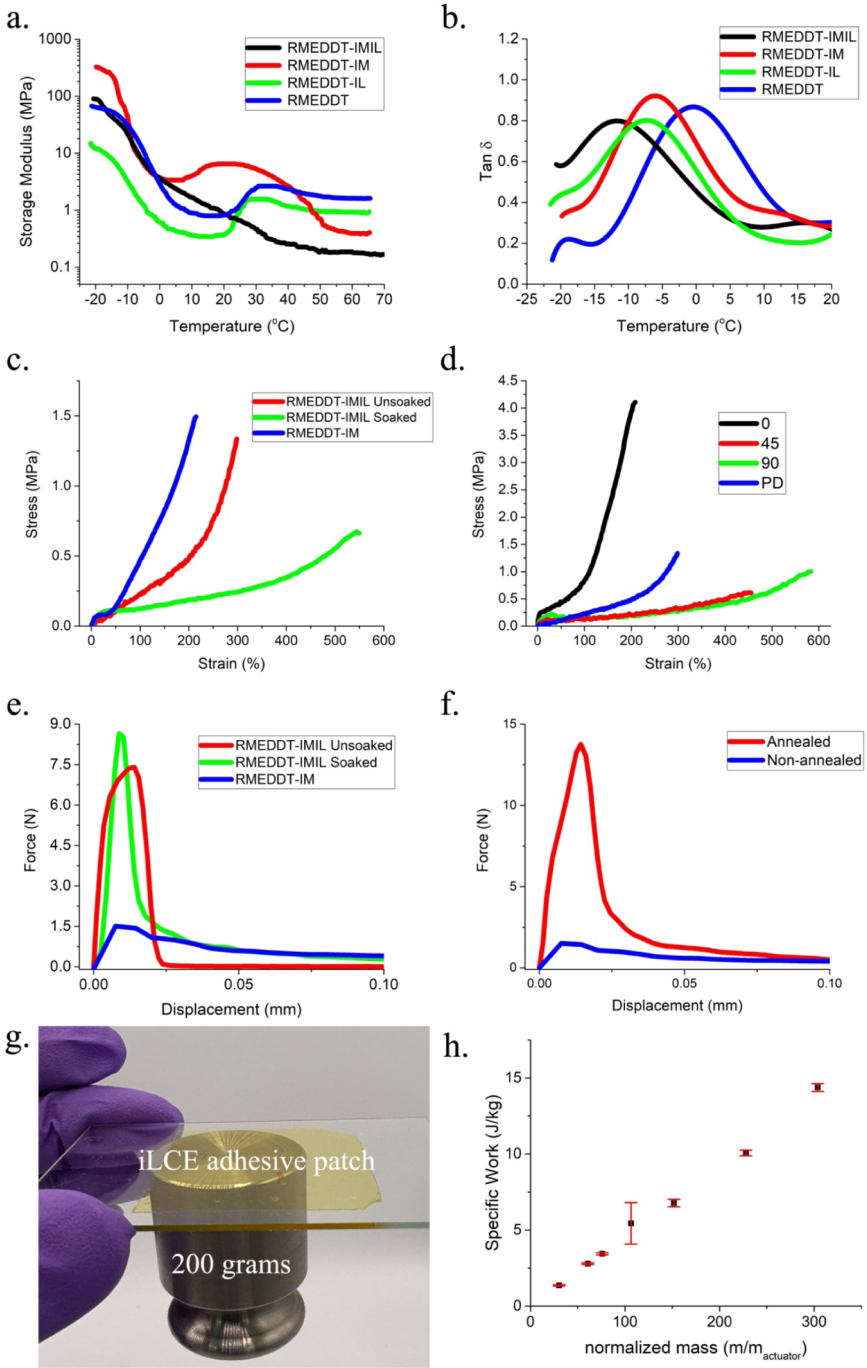
(a) Storage modulus versus temperature and (b) tan δ curves of the compositions from Table 1. (c) Tension test: Stress–strain curves comparing RMEDDT-IMIL (soaked and unsoaked) to RMEDDT-IM in the polydomain state and (d) as a function of the director orientation (90°, 0°, and 45°) of RMEDDT-IMIL films with respect to the tensile axis. Polydomain (PD) samples are overlaid for comparison. (e) Adhesion test: force–displacement plot of probe tack test, under 5 N loading conditions, comparing polydomain RMEDDT-IM and RMEDDT-IMIL (soaked and unsoaked). (f) Force–displacement measurements from a probe tack test showing the magnified adhesion because of thermal treatment (annealed) versus that without heat treatment (nonannealed) on polydomain samples. (g) Adhesion of an iLCE film that demonstrates lifting a 200 g weight. (h) Specific work density of monodomain RMEDDT-IMIL film measured by characterizing the tensile displacement of a suspended mass. Weights ranging from 2 to 15 g were used, with the force from gravity acting along the nematic director. The mass of the suspended load was normalized with respect to that of the iLCE.

Additional tensile tests were performed on molecularly ordered films with different planar alignments with respect to the tensile axis, as shown in Figure 2d. iLCE shows significant anisotropy in mechanical properties. The strain at rupture decreases by ∼3× from 600 ± 50% for 90° samples to 190 ± 35% for 0° samples. Toughness was found to be 2.30 ± 0.55 MJ/m^3^ and 2.90 ± 1.50 MJ/m^3^, respectively. When the nematic director is at 45° to the tensile axis, the fracture strain was measured to be 430 ± 30%, with a toughness of 1.20 ± 0.20 MJ/m^3^.

The ability to broadly modulate the liquid crystalline order and mechanical responses can unlock new design spaces in their utilization as adhesives—an emerging area of applications for LCEs.^56^ Probe tack adhesion tests were performed to further understand this behavior within the context of doping iLCEs with the IL, which acts as a plasticizer and increases chain mobility (Figure 2e). The stress–strain behavior in Figure 2c outlines this increase in chain mobility, which is evident from the magnification and lowering of the soft elastic plateau in the IL-soaked RMEDDT-IMIL. This contributes to a significantly magnified adhesion response. The force–displacement results from the adhesion test without any heat treatment (nonannealed case) are summarized in Figure 2e. The adhesion strength was characterized from the peak force by using the contact area of the probes with the iLCE (lateral dimensions of the sample). Here, RMEDDT-IM displays a small maximum adhesion strength of 20.1 ± 5.9 kPa, whereas the force for RMEDDT-IMIL (unsoaked) displays a larger adhesion strength of 61.7 ± 20.3 kPa. When RMEDDT-IMIL films are soaked, there is a nominal increase in the adhesion strength, yielding a value of 74.9 ± 29.8 kPa. iLCE doped with IL shows potential for utilization as pressure-sensitive adhesives. LCEs are known to form intimate contact when subjected to thermal cycling above the *T*_ni_,^56,68^ in addition to presenting adhesion that is sensitive to rate/stimuli^69–71^ and nematic order.^72^ Figure 2f explores this as an orthogonal strategy to magnify the adhesion of iLCE using the RMEDDT-IM system. Here, the sample was subjected to heat treatment and found to show a higher adhesion strength of 125.8 ± 38.1 kPa in comparison to the nonannealed case from Figure 2e. This approach was used to illustrate the ability to adhere a 200 g weight to the iLCE sample, where it is immobilized against gravity (Figure 2g).

The mechanically tough iLCE, which can potentially be integrated with suspensory structures using pressure-sensitive adhesion, can offer a platform material for work-dense actuation. This was explored in Figure 2h, which illustrates the specific work generated by RMEDDT-IMIL. The mass-specific work density was found to monotonically increase with increasing mass, consistent with prior observations.^14^ In response to a tensile load applied by the suspended weight, the actuator was found to lift ∼300× its own weight while generating a maximum work density of ∼14.4 J/kg. Application of loads larger than this led to the fracture of the iLCE samples under tension. The work-density of LCE in prior studies is ∼2 J/kg.^14,73^ Liquid crystalline systems synthesized using “isotropic genesis” have demonstrated higher work densities (50 J/kg), albeit at significantly higher actuation temperatures (150 °C), when the actuation profiles were optimized by controlling the ratio of the diacrylate mesogens to the amine chain extenders.^74^ High-performance photoresponsive LCEs have scaled the work content to >200 J/kg (over 200 kJ/m^3^).^75^ While more modest in work content than these LCEs, the iLCE presents a pathway to realize useful work densities with *T*_ni_ values in a biocompatible temperature range.

### 3.2 Self-Sensing with iLCE

iLCE actuates upon heating, but does its electronic conductivity cross-couple with the thermotropic response? To explore this, the electrical resistance through the thickness of an 80 μm thick iLCE film was measured as a function of temperature. The film was sandwiched between ITO-coated glass slides and subjected to heating/cooling cycles on a hot plate (Figure 3a,b). We found that the resistivity of the sample decreased by ∼75%, dropping from 412.5 ± 31.0 kΩ**·**cm at room temperature to 95.8 ± 3.8 kΩ**·**cm at 115 °C. This is characteristic of the Vogel–Fulcher–Tammann behavior, where the segmental mobility of the network defines the conductivity in ionic polymers.^76^ The observation of this effect within the thermomechanical actuation window of the iLCE enables self-sensing.

**Figure 3:**
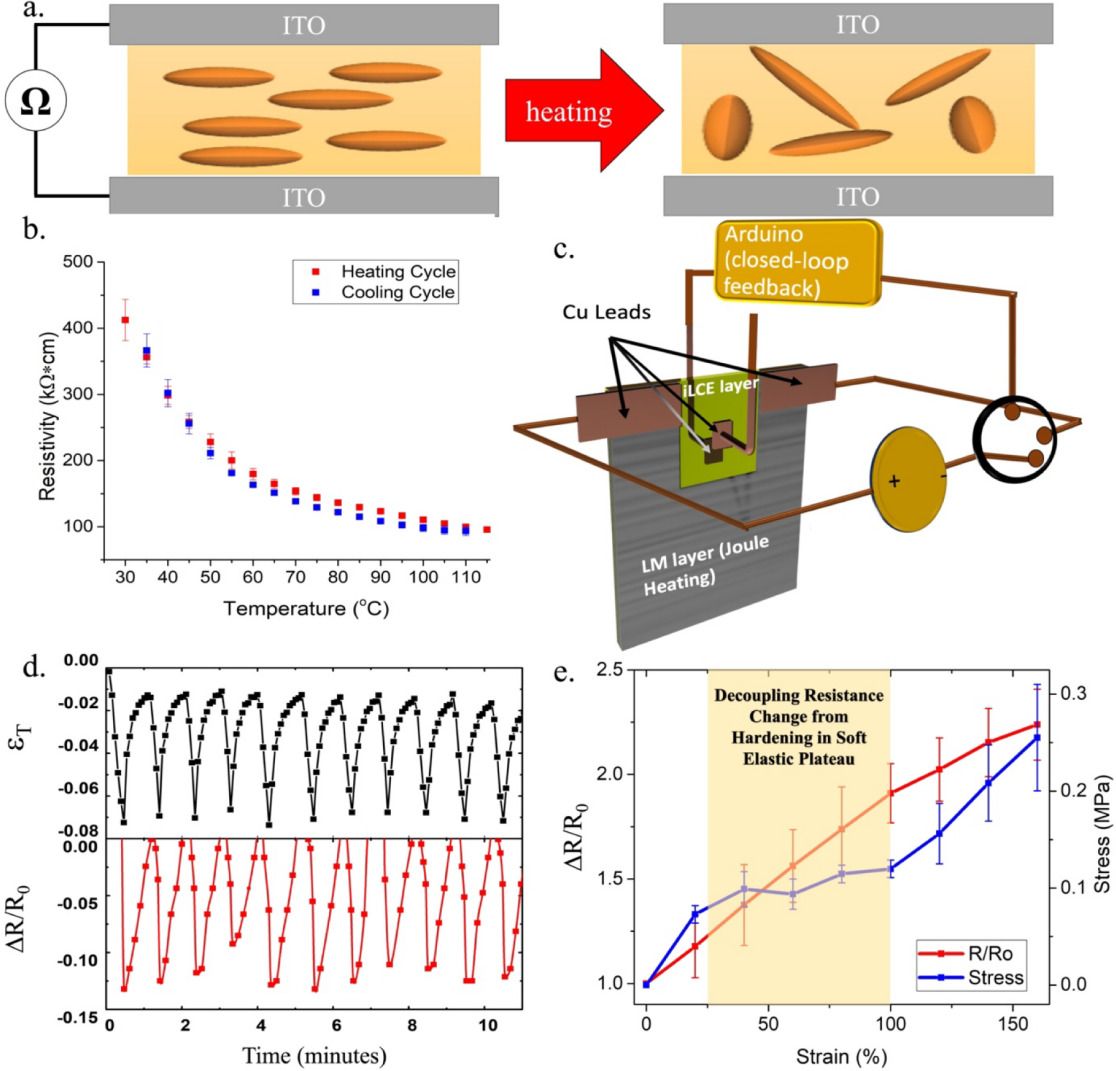
(a) Schematic of the setup used to measure the electrical conductivity, where the iLCE is sandwiched between two ITO coated slides and heated. (b) The change in electrical resistivity of the RMEDDT-IMIL film was measured as a function of temperature. (c) Schematic of the iLCE self-sensing unit, RMEDDT-IMIL coated with LM, which actuates the iLCE via Joule heating. In the uncoated region of the iLCE, Cu electrodes were attached to both sides of the sample and used to measure the resistance through the thickness. The change in the electrical resistance of the iLCE during its actuation was used to modulate the delivery of the electrical power for Joule heating. (d) Stacked plots showing the relative change in resistance (bottom) and its correlation with the strain (top) induced from the electrothermal self-sensing capability of the iLCE in a closed-loop configuration system. See Video S1 for the actuation cycles. (e) The change in resistance was measured as a function of strain and overlaid with the stress response of RMEDDT-IMIL in samples with the director that is oriented at 90° to the tensile axis. The highlighted region represents the decoupling of electrical resistance from changes in mechanical impedance (strain hardening) within the soft-elastic plateau.

Upon realizing that iLCE is a thermally responsive material that can sense its temperature, it was harnessed to create a feedback-loop system. An RMEDDT-IMIL iLCE film was coated with a layer of eutectic gallium–indium liquid metal (LM) over a U-shaped region (Figure 3c). The LM was synthesized by combining raw gallium and indium in a 3:1 weight ratio. The LM layer was applied to the iLCE using a brush in a manner similar to that in ref 77. This LM layer served as a flexible Joule heater when electrically powered. To achieve resistive feedback during the thermomechanical actuation of the iLCE, the uncoated region of the iLCE was sandwiched between two Cu plates. An Arduino-based control system was programmed to regulate the actuation strokes through current modulation by supplying a low voltage (<3 V) to the LM electrode based on the resistive feedback. The actuation process involved powering the Joule heater from the LM until a targeted change in resistance was measured through the thickness of the iLCE film. The power was then terminated by the microcontroller, and the sample was allowed to cool down and relax to its original state. The heating cycle was triggered again once the resistance increased to its original value, and the cycle continued. Figure 3d and Video S1 illustrate the actuation strain overlaid with the change in resistance measured through the thickness of the actuator. In this case, the film undergoes multiple cycles, where ∼7% strain is generated, and a ∼13% change in resistance was set as the threshold for terminating power to the LM Joule heater. The iLCE assimilates the capabilities achieved in systems that include LCE that contain IL in phase-separated domains.^9^ Harnessing this in a single-phase iLCE system can allow for their utilization in self-sensing actuators across length scales, including at finer scales. Furthermore, the iLCE may offer a higher work-density resulting from the entirety of the material generating the actuation, in contrast to a phase-separated mixture that contains active and passive (IL) domains. Thus, iLCEs present a pathway for assimilating sensing and electrothermal actuation within a single unit to enable a closed-loop system.

As illustrated in Figure 2d, RMEDDT-IMIL samples that are subjected to tension at 90° to the nematic axis demonstrate a pronounced soft elastic plateau. In this regime, the material is capable of large strain deformation (∼100%) without significant hardening. This led us to explore whether there is a cross-coupling between the mechanical response in the soft elastic plateau and the electronic properties. The ability to transduce shape change in this regime into a change in electrical resistance offers a framework for strain sensing without a change in the mechanical impedance. Figure 3e shows the change in resistance of an iLCE as the film is strained orthogonal to its nematic axis and overlaid with the measured stress. Within the ∼25 to 100% strain region, the material has a flat stress–strain curve (soft elastic plateau), while the resistance is shown to maintain a linear increase. This observation suggests that iLCE films as strain sensors can eliminate back-coupling of the strain measurement to the constitutive response of the host structure when embodied with soft robotics or biomedical systems. The sensor can stretch to large strains without any appreciable hardening within the soft elastic plateau.

### 3.3 Ionic Actuation with iLCE

We further explored the voltage-induced migration of ions in the iLCE system to drive athermal mechanical actuation. In doing so, the role of molecular anisotropy in the bending was explored. The RMEDDT-IMIL films, which were subsequently soaked with IL, were spin-coated with PEDOT:PSS electrodes. Ionic actuation in iLCE films was studied by applying 1 and 3 V at the electrodes. The films bend away from the anode, which indicates that the mobility and accumulation of the anions drive the ionic actuation (Supplementary Videos S2–S5). Tensile strains are generated at the anode. The alignment orientation of the monodomain (planar-oriented) iLCE was varied between 0°, 45°, and 90° with respect to the long axis of strip-like samples. Figure 4a–d shows the strain/displacement of bending of each orientation as a function of time with the application of a square wave voltage (8.33 mHz). It is apparent that molecular anisotropy defines the magnification of the bending strains generated with iLCE. The introduction of charged moieties into the backbone of the iLCE produces larger strains (2× higher) in comparison to the IL-doped LCE.^49^ This likely results from the efficient ion transport pathways in the iLCE system, in contrast to those possible in the phase-separated IL-doped LCE. This strain response also outstrips that observed recently with ionic liquid crystals that are composited with poly­(vinyl alcohol).^78^ A comparison of the actuation profiles to PD iLCE shows the role of molecular anisotropy in magnifying strain generation from ion migration. The PD sample (Figure 4d) was created by cross-linking oligomeric inks devoid of any nematic order, as they were not subjected to alignment via stretching. Figure 4e illustrates snapshots of the actuation for a range of offset angles of *n*° with respect to the long axis of the cantilevers. The samples with the nematic director at 45° to the long axis of the cantilever illustrate a native twist along their axis. This is likely a result of a polymerization/cross-linking gradient during sample preparation. No change in the twist is observed during ionic actuation.

**Figure 4:**
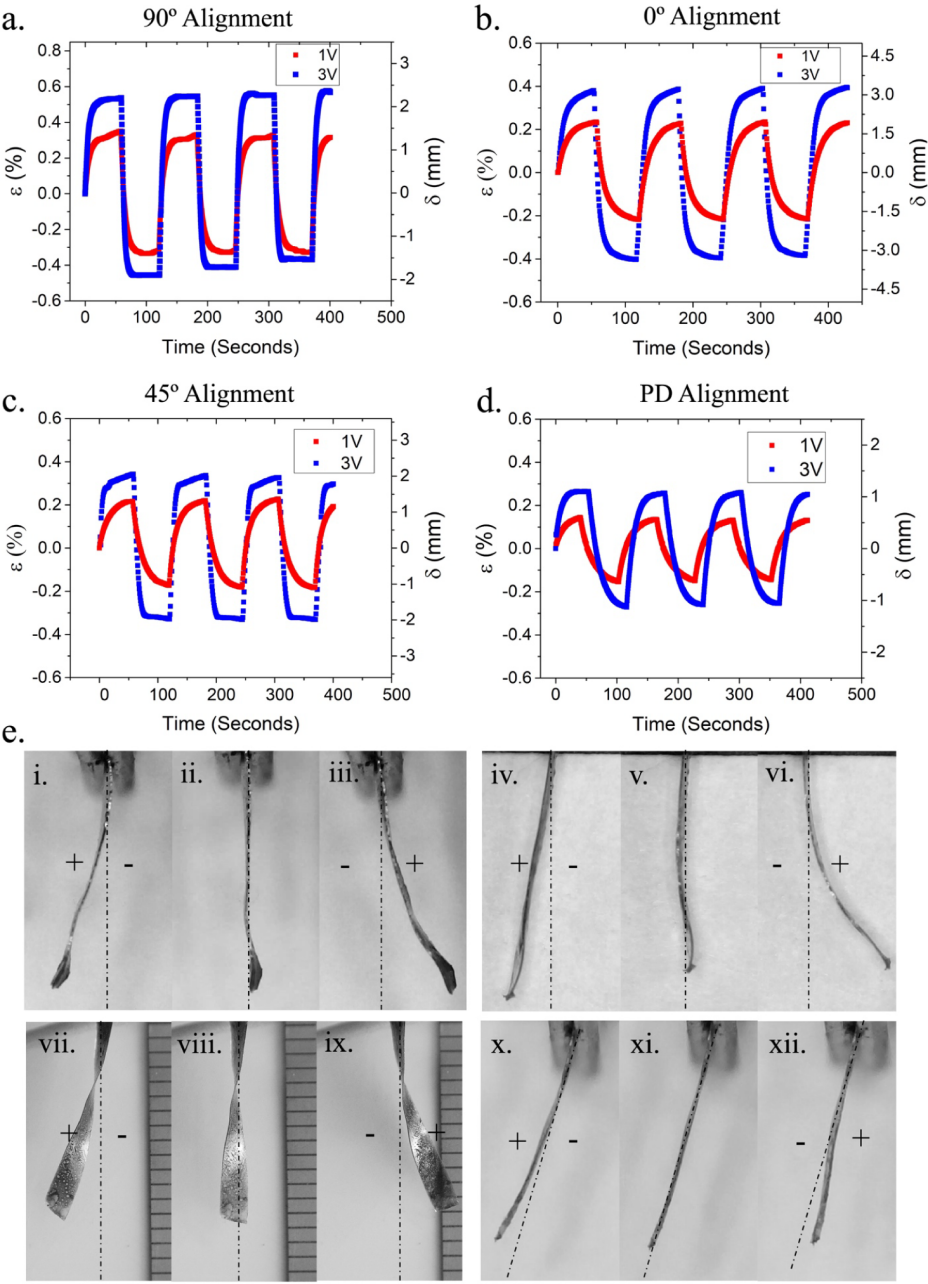
(a–d) Plots of athermal ionic actuation cycles: strain and tip displacement versus time for (a) 90°, (b) 0°, and (c) 45° alignment of the nematic director with respect to the long-axis of the actuator. (d) Actuation response of the polydomain (PD) iLCE. (e) Images show snapshots of the bending of RMEDDT-IMIL toward the cathode during repetitive actuation and switching of the polarity. This is shown for of 90° (i–iii), 0° (iv–vi), 45° (vii–ix), and PD (x–xii), where samples were actuated at 3 V.

Figure 5a shows the peak-to-peak bending strain values (ε_p_) for the samples illustrated in Figure 4a–d. For 1 V actuation cycles, ε_p_ for 0°, 45°, and 90° is 0.45 ± 0.04%, 0.40 ± 0.01%, and 0.66 ± 0.02%, respectively. For 3 V, the magnitude increased to 0.79 ± 0.01%, 0.66 ± 0.00%, and 1.01 ± 0.06% for each respective orientation. The PD sample at 1 and 3 V had ε_p_ values of 0.28 ± 0.01% and 0.52 ± 0.01%. Clearly, the actuation of iLCE via ion migration is magnified by the molecular order. The ε_p_ for all the orientations of the director (0°, 45°, and 90°) produces ∼2× larger ε_p_ than that for the PD sample. Also, in the 90° sample, where the *n* is transverse to the sample (along the short axis), the largest peak-to-peak ε_p_ bending strains >1% are generated. Clustering of anions will lead to volumetric expansion in the vicinity of the anode. However, the anisotropy in the mechanical properties due to the molecular order results in greater expansion transverse to the molecular director than along it. This anisotropy is evident in Figure 2d. These tensile strains transverse to the director magnify the apparent bending strains in the 90° case, which likely underpins the observations in Figure 5a. It is currently unclear to us why the 45° sample produces a smaller bending strain than the 0° sample. However, the lack of order in the PD sample in Figure 5a presents substantially degraded actuation. This highlights the role of nematic order in magnifying the electromechanical performance of iLCE. The electromechanical performance of the iLCE is compared to other ionic actuator systems. Figure 5b illustrates the peak-to-peak bending strains of iLCE actuators, which shows their performance in comparison to other optimized material systems. Opportunities can emerge from the optimization of the iLCE using design ideas from state-of-the-art ionic actuators, including the design of higher-performance electrodes and interfaces, to magnify this performance.

**Figure 5:**
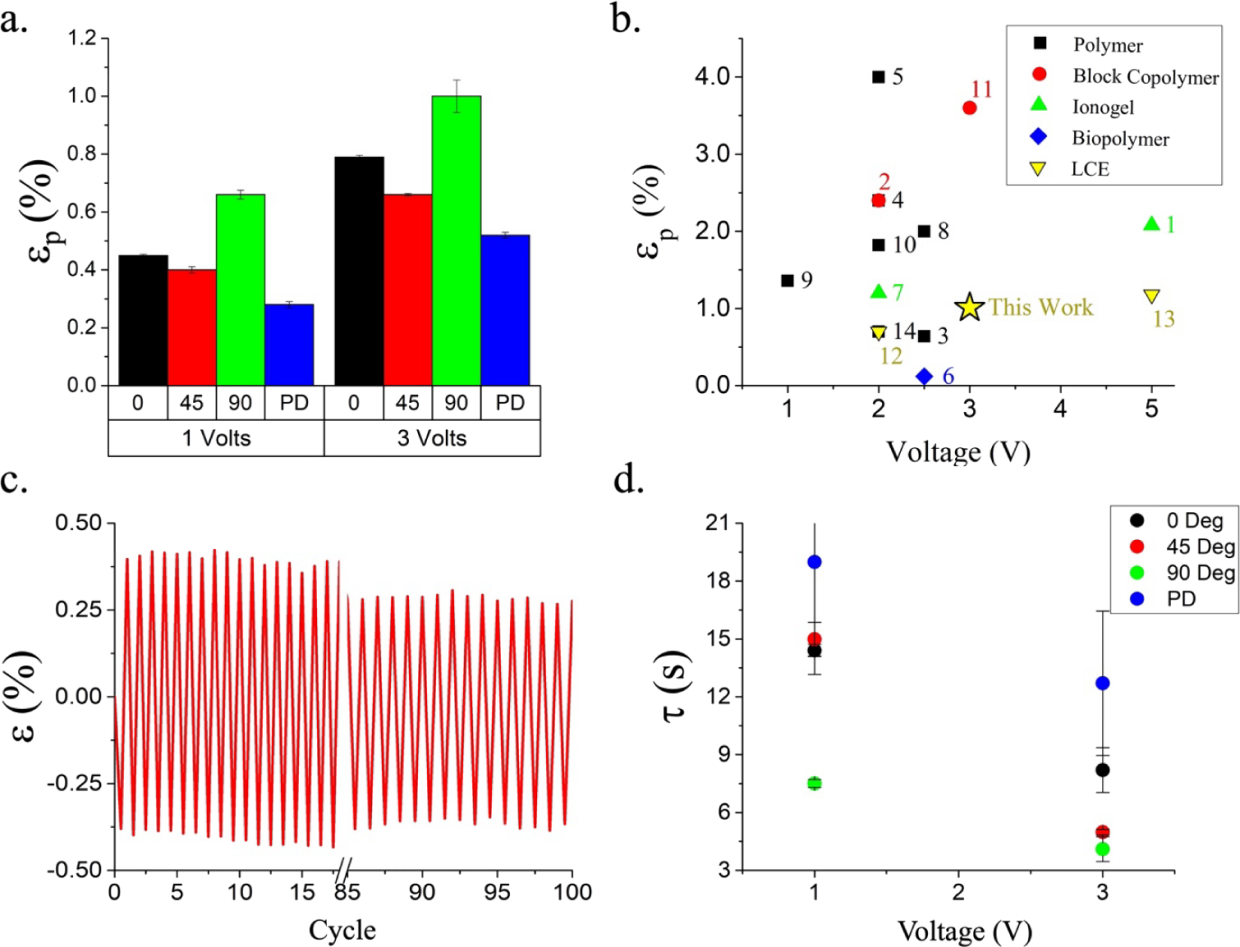
(a) Plot of maximum peak-to-peak (ε_p_) strain for each iLCE sample (0°, 45°, 90° offset angle and polydomain (PD)). (b) Peak-to-peak strains versus applied voltage comparing ionic bending actuators from the literature to the current work. The references corresponding to the various classes of actuators that are numbered from 1 to 14 are listed in Section S6.0. The “Polymer” class of actuators consists of examples from the literature that do not neatly fit into the other classes. (c) Repeatability of iLCE actuation is shown for 100 actuation cycles. (d) Time constant (τ) as a function of voltage for each sample is shown (0°, 45°, 90° offset angle, and PD).

A 90° sample was subjected to a square wave impulse at 16.67 mHz and actuated 100 times at 3 V, as shown in Figure 5c. At the higher frequency, the peak-to-peak strain declined to ∼0.8%. Also, with progressive actuation cycles, the peak-to-peak strain declined by ∼0.15 ± 0.01%. This was calculated by comparing the first 15 cycles and the last 15 cycles. This decline is traceable to the current limitations of iLCE in enabling continuous pathways for ion transport, in comparison to that observed in say, sulfonated block copolymers.^47^ The accumulation of the bending strains during the application of voltages is defined by the mobility of the ions. The time constants for the evolution of the bending strain (τ) for the actuation cycles in Figure 4 were calculated by fitting the data to an exponential function $\left(\varepsilon =Ae^{-t/\tau }+\varepsilon_{0}\right)$ , where *A* is a pre-exponential constant, ε_0_ is an offset term, and τ is the time constant. At 1 V, the τ values for 0°, 45°, and 90° were found to be 14.4 ± 0.3, 15.0 ± 0.9, and 7.5 ± 0.2 s, respectively. At 3 V, the τ values for 0°, 45°, and 90° were found to be 8.2 ± 1.2 s, 5.0 ± 0.1 s, and 4.1 ± 0.6 s, respectively (Figure 5c). For the PD sample, at 1 and 3 V, the time constants were found to be 19.0 ± 5.8 and 12.7 ± 3.7 s, respectively. The actuation was found to be the most robust for the samples with the nematic direction orthogonal to the long axis of the cantilever and that of ion transport (90° samples). Higher voltages led to the expected acceleration of actuation rates and smaller τ. Also, the PD samples devoid of uniaxial molecular order presented the most sluggish actuation (highest τ) while also generating the smallest strains (Figure 5a,d). While all the films used in the electromechanical experiments were soaked in additional IL, broadband dielectric spectroscopy of RMEDDT-IMIL showed no statistical difference in the ionic DC conductivity, as shown in Figure S5 and discussed in Section S5.0.

## 4 Conclusions

iLCEs containing cationic groups in their backbone exhibit mechanical responses to stimuli, including temperature and electric fields. They present broadly tunable thermomechanical and electromechanical properties as a function of the ionic content—both in the backbone and with IL dopants. The thermotropic responses of iLCEs are tunable via the introduction of IL, which is found to lower the temperature of actuation to ∼40 °C. The iLCEs (like conventional LCEs) also generate contractile strains parallel to the nematic director upon heating. IL dopants further tune the mechanical properties of the iLCEs to endow them with higher toughness. The iLCEs doped with IL with their pronounced soft elasticity show promise as pressure-sensitive adhesives.

Multifunctional responses in iLCE also emerge due to the cross-coupling of their responses to thermal, electrical, and mechanical stimuli. The iLCE doped with IL demonstrates an electrical resistance that is dependent on temperature. A decline in conductivity coincides with the thermotropic actuation window, where actuation strains >30% and work densities >14 J/kg are achieved. This enables a framework where iLCE can self-sense its state during actuation. This is used in a prototypical configuration, where the iLCE serves as both the muscle and the temperature sensor. Beyond the thermomechanical response, the coupling of the soft elasticity nascent to iLCE with its electronic conductivity presents a framework for large strain sensing. The large deformability at the constant stress plateau presents a linear change in electronic resistance, but with the iLCE itself undergoing no strain hardening. iLCE also actuates by bending in response to ion gradients generated with low voltages (≤3 V). Bending strains are found to compare favorably with those achieved with conventional ionic actuators. Furthermore, the actuation profiles are a function of the orientation of the nematic director. The largest actuation strains and actuation rates emerge when the nematic director is orthogonal to both the direction of ion migration and the long axis of the cantilever.

The ability of iLCE to assimilate multifunctional responses presents a pathway for realizing a new class of electroactive polymers, where sensing and actuation can be nascent material responses. Progressive optimization of this material system, where these responses can be further magnified, holds implications for broader classes of iLCE, where actuation, mechanical properties, and cross-coupling between electromechanical and thermomechanical properties can be directed via patterning of the molecular director using 2D blueprinting and 3D printing strategies.

## Supplementary Material













## Notes

Synthesis methods; NMR characterization; gel fraction and FTIR measurements; WAXS characterization; broadband dielectric spectroscopy; and table of references corresponding to the data points illustrated in Figure 5b (PDF)
Self-regulated actuation with iLCE (Video S1) (MP4)
Bending of an iLCE sample with director aligned at 90° to the long-axis of the cantilever (Video S2) (MP4)
Bending of an iLCE sample with director aligned at 0° to the long-axis of the cantilever (Video S3) (MP4)
Bending of an iLCE sample with director aligned at 45° to the long-axis of the cantilever (Video S4) (MP4)
Bending of a polydomain iLCE sample (Video S5) (MP4)
