# Supplementary material for: Multifunctional Ionene Liquid Crystal Elastomers
Source: ACS Appl Mater Interfaces. 2025 Jul 17;17(30):43620–32. doi: 10.1021/acsami.5c08503 (PMC12314865; doi:10.1021/acsami.5c08503)
Supplement: Supplementary file 1 [file am5c08503_si_001.pdf]

Supporting Information

Multifunctional Ionene Liquid Crystal Elastomers

Zachary Kuzel[1], Arul Clement[1], Mohsen Tabrizi[1], Abdullah AboHussien[1], Sivakumar Irla[1], Hassan Beheshti Seresht[1], Youngjae Chun[1,2,3], Stephanie Tristram-Nagle[4], Qihan Liu[5], and M. Ravi Shankar[1,5] *

1

Department of Industrial Engineering

Swanson School of Engineering

3700 O'Hara Street,

University of Pittsburgh

Pittsburgh, PA 15261

2

Department of Bioengineering

Swanson School of Engineering

3700 O'Hara Street,

University of Pittsburgh

Pittsburgh, PA 15261

3

McGowan Institute for Regenerative Medicine

450 Technology Drive

University of Pittsburgh

Pittsburgh, PA 15219

[4]

Biological Physics Group, Physics Department,

5000 Forbes Avenue,

Carnegie Mellon University, Pittsburgh, PA 15213

[5]

Department of Mechanical Engineering and Materials Science

Swanson School of Engineering

3700 O'Hara Street,

University of Pittsburgh

Pittsburgh, PA 15261

* ravishm@pitt.edu

1.0 Reaction schematic of diallyl imidazolium

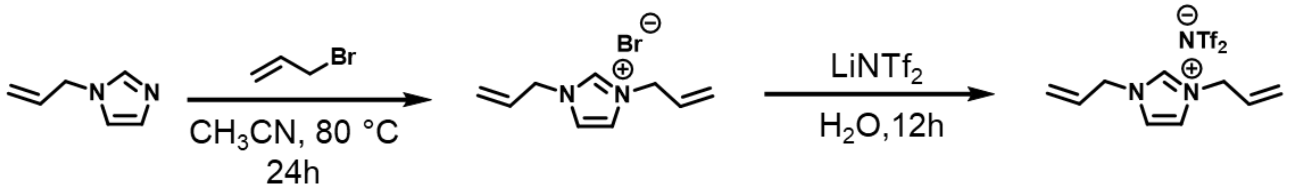

Figure S1: Synthetic scheme of 1,3 diallylimidazolium bis (trifluromethylsulfonyl)imide

2.0 NMR of diallyl imidazolium

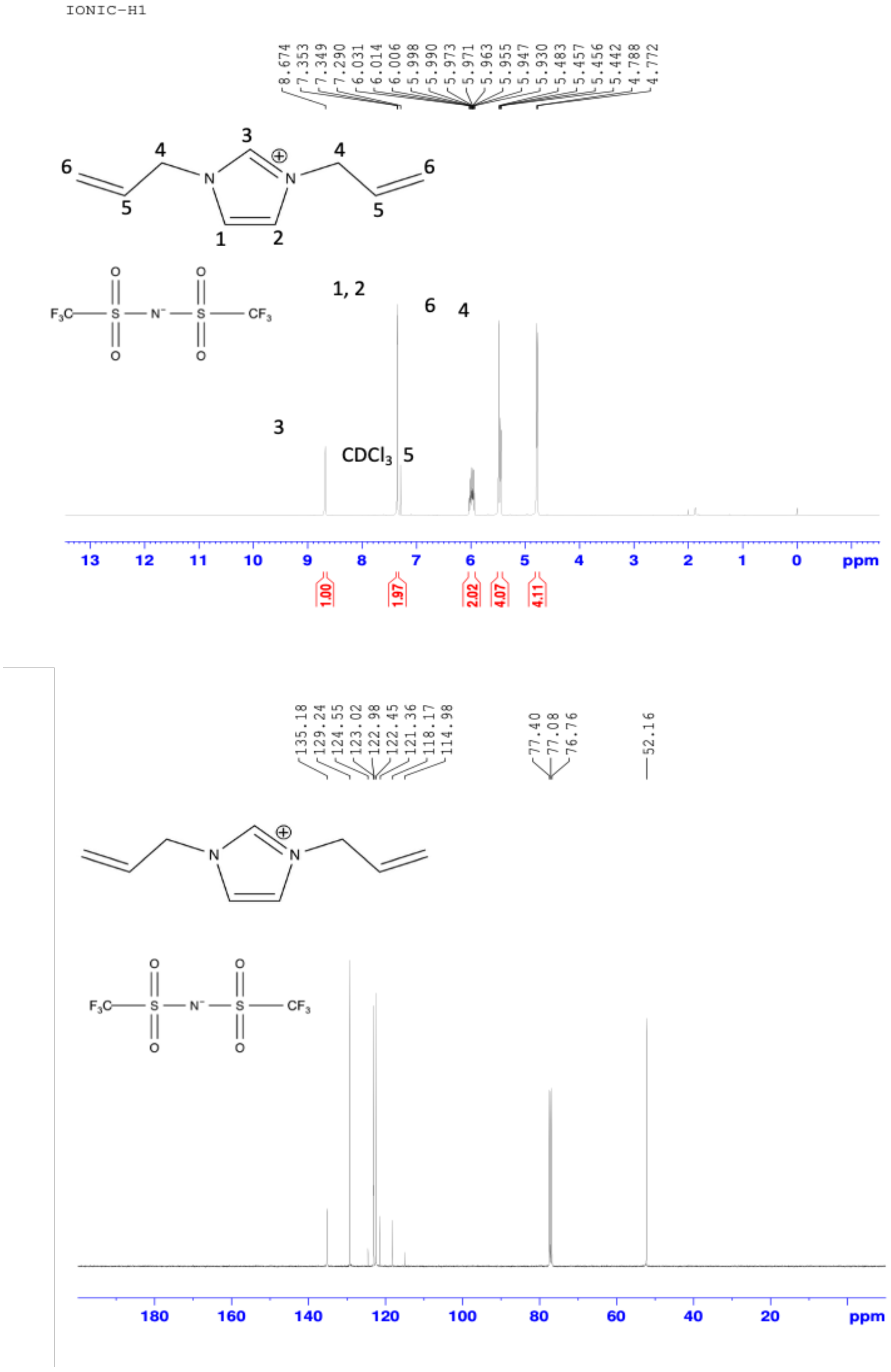

Figure S2: ^{1}H (top) and ^{13}C-NMR (bottom) spectra of imidazolium crosslinker

3.0 Gel Fraction and FTIR Methodology

To quantify the total extent of crosslinking reactions from the acrylate and alkene reactive groups, rectangular iLCE films without ionic liquid were subjected to full immersion in chloroform for 24 hours at room temperature. After the 24-hour period, the films were removed from the solvent and dried in a vacuum for another 24 hours at room temperature. The gel fraction of the elastomer was then calculated from the masses of the films before and after swelling was conducted, using the following relationship:

$$\text{GF \%} = \frac{m_f}{m_i} \text{ x } 100, \qquad (S1)$$

where m_i and m_f are the respective initial and final masses of the film. A sample size of three films (n=3) were used in this experiment.

A Perkin Elmer FTIR spectra was used for further insight into the polymerization of the imidazolium species. To confirm the reaction of the imidazolium crosslinker, FTIR was performed on RMEDDT-IMIL films in both the pre-crosslinked oligomer state and the crosslinked elastomer state.

3.1 FTIR Results and Gel Fraction

FTIR results shows the vinyl peak in the IM monomer curve occurring at ~1645 cm^{-1}. RMEDDT-IMIL oligomer samples were analyzed using FTIR upon the completion of the step-growth thiol reactions. The oligomers revealed a more significant peak at a wavelength of ~1635 cm^{-1},

suggesting that a significant portion of vinyl groups reacted with thiol reactive groups along with the acrylate-thiol reactions. The elastomer confirms the disappearance of any distinct peaks, and gel fraction (GF) analysis further supports this theory. The gel fraction value, GF, was found to be 80% ± 1%. Ware has previously reported an LCE with a thiol-ene terminated chemistry that has a GF of 85% ± 4%[1], suggesting the iLCE's GF was reasonable and most monomers were reacted.

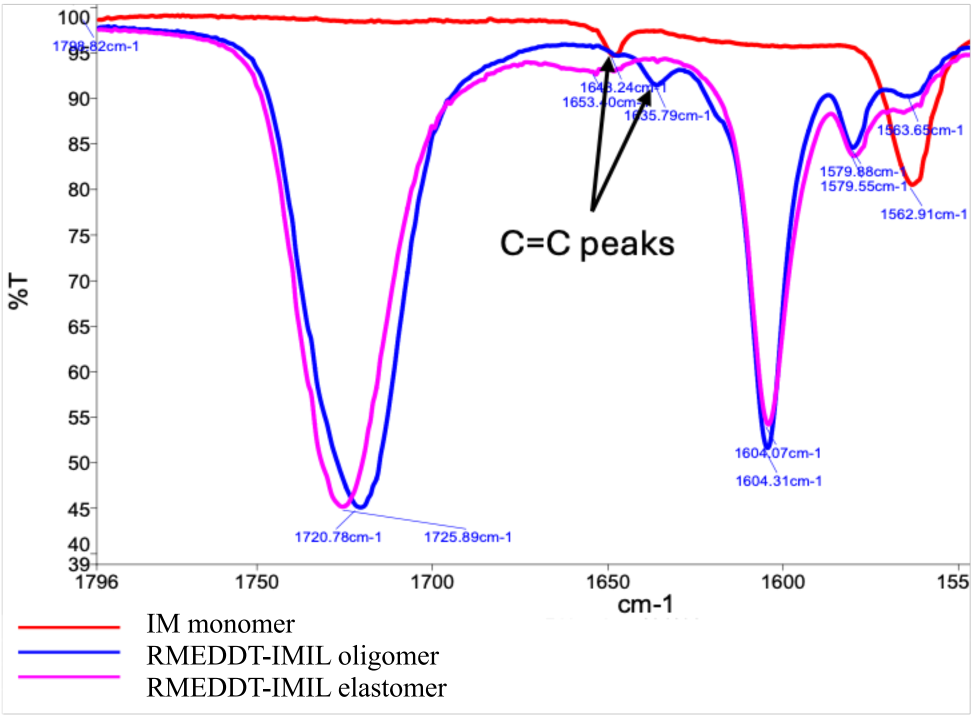

Figure S3: FTIR results of the RMEDDT-IMIL samples in the polymerized state, the oligomerized state, and a curve that analyzes only the diallyl imidazolium.

4.0 WAXS Methodology

Methods for the wide angle x-ray experiments and the data processing to derive the order parameters are identical to previous work done on LCEs[2]. WAXS scattering from a well-ordered iLCE or LCE sample is captured and rotated 90° clockwise, shown in columns **i.** and **ii.** in figure

S4. The images from column **ii.** are used to perform the data analysis as described in Mills et al[3]. Starting at the equator, integration is performed on the WAXS intensity in C vs. q_r in 10-degree π sectors in φ to construct a sector plot. The interchain D-spacing is determined by taking the greatest intensity, q_r, value and plugging it into the following equation

$$\frac{2\pi}{q_r} = D. \qquad (S2)$$

Subsequently, over a specified q_r range (often 0.8 to 2.2 $Å^{-1}$), the intensity as a function of φ is integrated (solid black circles). The detailed analysis and several other fitting parameters are laid out by Mills in ref [4].

4.1 WAXS Results

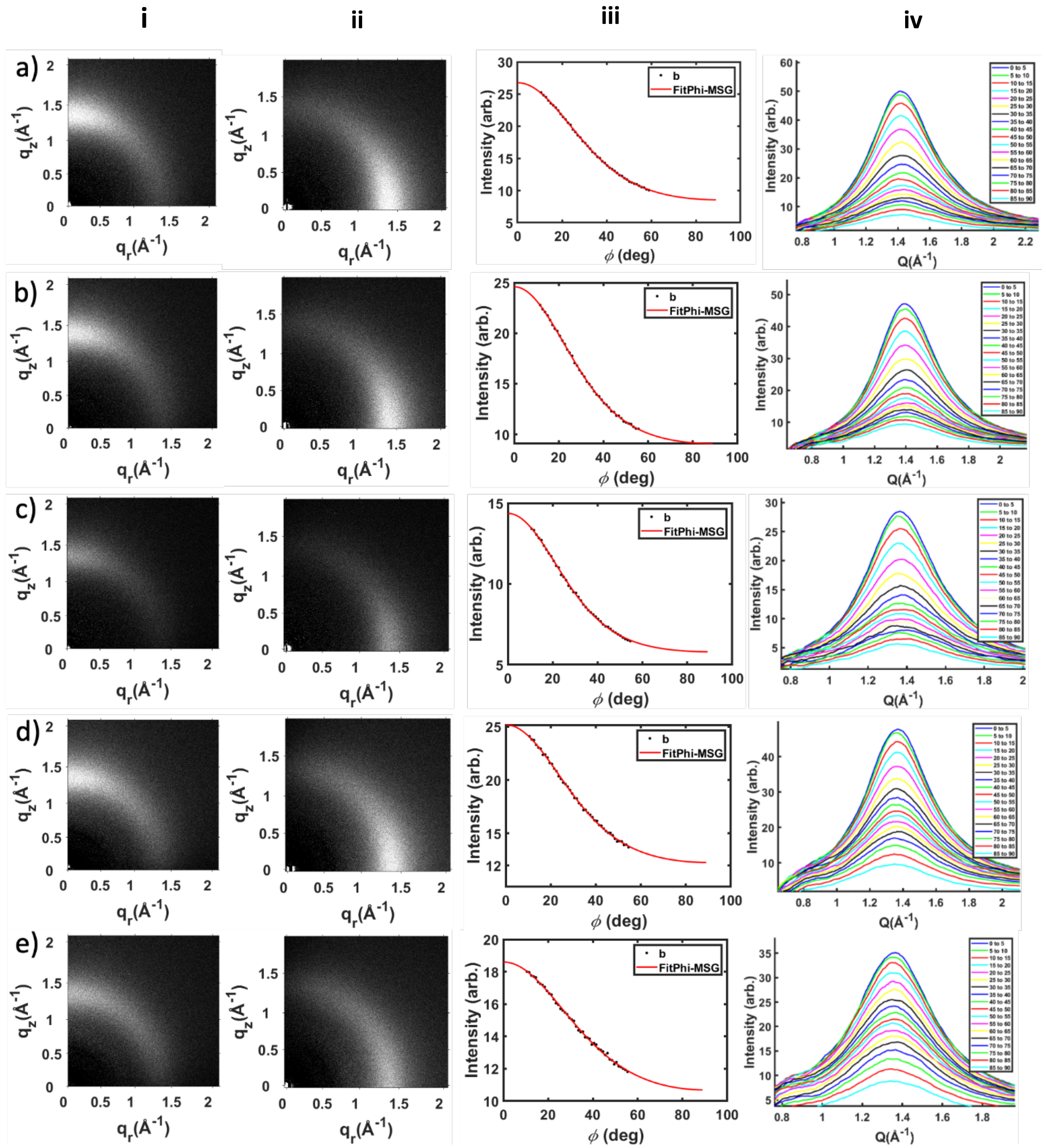

Figure S4: Data collection and analysis for WAXS of a) RMEDDT, b) RMEDDT-IL, c) RMEDDT-IM, d) RMEDDT-IMIL (unsoaked), and e) RMEDDT-IMIL (soaked).

5.0 Broadband Dielectric Spectroscopy: Conductivity Measurements

Conductivity is measured using a broadband dielectric spectroscopy (BDS), which places the sample as a capacitor between two parallel brass electrode plates. Ionic DC conductivity is defined as the plateau region of the real component of the conductivity. The flat conductivity regime is within the range of ~10^2 Hz to ~10^4 for the ionic samples that were tested. The plots below show the raw results from the BDS measurements. Statistical analysis between three samples for each case revealed statistical annulment of the initial hypothesis.

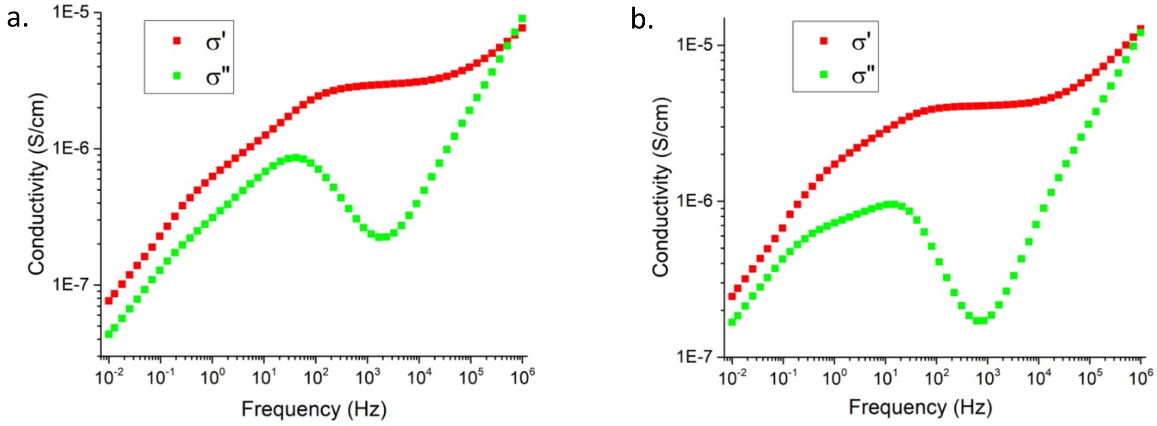

Figure S5: Representative plots from conductivity measurements from broadband dielectric spectroscopy showing the real (σ’) and imaginary (σ”) component of the conductivity for (a.) RMEDDT-IMIL and (b) RMEDDT-IMIL after soaking in ionic liquid.

6.0 Reference Table for Figure 5b

Ref. No.	Voltage	Strain	Membrane	Reference
1	5	2.08	Ionogel	Yan, Y., et al. (2017). Advanced Materials 29(23): 1606109.
2	2	2.40	Block Copolymer	Lee, J.-W., et al. (2013). Journal of Materials Chemistry C 1(24): 3784-3793.
3	2.5	0.64	Polymer	Wang, D., et al. (2017). RSC advances 7(50): 31264-31271.
4	2	2.40	Polymer	Festin, N., et al. (2013). Smart Materials and Structures 22(10): 104005.
5	2	4.00	Polymer	Zhou, D., et al. (2003). Electrochimica acta 48(14-16): 2355-2359.
6	2.5	0.12	Biopolymer	Wang, F., et al. (2016). Soft Matter 12(1): 246-254.
7	2	1.20	Ionogel	Takeuchi, I., et al. (2009). Electrochimica acta 54(6): 1762-1768.
8	2.5	2.00	Polymer	Wu, G., et al. (2019). Advanced Materials 31(25): 1806492.
9	1	1.36	Polymer	Umrao, S., et al. (2019). Science Robotics 4(33): eaaw7797.
10	2	1.82	Polymer	Monobe, H., et al. (2020). Japanese Journal of Applied Physics 59(SD): SDDF08.
11	3	3.60	Block Copolymer	Kim, O., et al. (2016). Nature Communications 7(1): 13576.
12	2	0.70	LCE	Feng, C., et al. (2019). Macromolecular rapid communications 40(19): 1900299.
13	5	1.18	LCE	Deng, Y., et al. (2024). Advanced Functional Materials 34(40): 2403892.
14	2	0.70	Polymer	Cao, S., et al. (2022). ACS Applied Materials & Interfaces 14(38): 43701-43710.

References

(1) Saed, M. O.; Ambulo, C. P.; Kim, H.; De, R.; Raval, V.; Searles, K.; Siddiqui, D. A.; Cue, J. M. O.; Stefan, M. C.; Shankar, M. R. Molecularly-engineered, 4D-printed liquid crystal elastomer actuators. *Advanced Functional Materials* **2019**, *29* (3), 1806412.

(2) Skandani, A.; Clement, J. A.; Tristram-Nagle, S.; Shankar, M. R. Aliphatic flexible spacer length controls photomechanical response in compact, ordered liquid crystalline polymer networks. *Polymer* **2017**, *133*, 30-39.

(3) Mills, T. T.; Toombes, G. E.; Tristram-Nagle, S.; Smilgies, D.-M.; Feigenson, G. W.; Nagle, J. F. Order parameters and areas in fluid-phase oriented lipid membranes using wide angle X-ray scattering. *Biophysical journal* **2008**, *95* (2), 669-681.

(4) Mills, T. T.; Tristram-Nagle, S.; Heberle, F. A.; Morales, N. F.; Zhao, J.; Wu, J.; Toombes, G. E.; Nagle, J. F.; Feigenson, G. W. Liquid-liquid domains in bilayers detected by wide angle x-ray scattering. *Biophysical journal* **2008**, *95* (2), 682-690.
